# Oleate of Copper in Tinea Capitis

**Published:** 1885-08-20

**Authors:** Robert Boal

**Affiliations:** Peoria, Illinois


### OLEATE OF COPPER IN TINEA CAPITIS.

BY ROBERT BOAL, M.D., PEORIA, ILL.

Among the parasitic skin diseases few are more annoying to the patient, or whose treatment is often so unsatisfactory and perplexing to the physician, than the affection popularly known as scald head. Its appearance is so familliar and well marked that any description of it is unnecessary. Nor can it be mistaken for any other affection. It is called by various names, trycophytosis capitis, tinea circinatis, tinea capitis, and others, all these names having reference to the parasite which produces these changes on the skin. The great difficulty in curing many cases largely depends upon the depth and extent to which the parasite has propagated itself. If its ravages are confined to the surface of the skin, and do not reach the hair follicles, any of the ordinary parasiticides will generally arrest the disease; or juniper tea, sulphur and other remedies of that class will suffice. But if the parasite has burrowed down in to the hair follicles, stronger remedies will be required. The milder agents which have been named may cause a temporary improvement by destroying the parasites upon the surface, while those that are under it and in the hair follicles are not reached. The remedy which I have found to most effectually destroy these deeply hidden parasites is the oleate of copper. My attention was called to this preparation some two years ago, in a paragraph in one of the medical journals, and I determined to give it a trial upon the first opportunity.

Last year I was called upon to see a patient, seventeen years of age, well formed, robust, and in apparent good health. I found the entire scalp covered with large, branny scabs, from beneath which a discharge had issued which became hardened by the contact of the air; the hairs were broken off, and looked like stubble. They had lost their glistening appearance, were dry, and apparently dead. The eruption not only covered the entire scalp, but extended down to the upper side of the face and over the ears. It was one of the worst and most unpromising cases I ever saw. Nearly all of the ordinary germicides had been tried without avail under other hands. I determined to use the oleate of copper. An ointment of cosmoline, containing 20 per cent. of the oleate, was applied twice a day, having previously gently removed all the detached and partially detached branny scabs with a hair brush.

Under this treatment the case began to improve, and at the end of three weeks the scalp assumed a healthy appearance, the hair grew rapidly, and the disease was cured. More than a year has elapsed since that time, and there is no return of the disease. The oleic acid with which the copper is combined seems to have the power to penetrate to the depth of the hair follicles laden with the copper, a combination which effectually destroys the parasite.

No constitutional treatment was used or required. The ointment should be well but gently rubbed upon the parts once or twice a day as required. In most cases an ointment containing from 10 to 15 per cent. of the oleate of copper will be strong enough. In my opinion, this is one of the most efficient remedies we possess in this troublesome and disagreeable affection. I shall use it in the future, as in the past, with more confidence than any other remedy, and I do not hesitate to recommend a trial of it to others.—*Peoria Med. Monthly.*

